# Lateral flow immunoassay for simultaneous detection of *C. difficile*, MRSA, and *K. pneumoniae*

**DOI:** 10.1007/s00604-024-06701-w

**Published:** 2024-10-01

**Authors:** Ana Rubio-Monterde, Lourdes Rivas, Marc Gallegos, Daniel Quesada-González, Arben Merkoçi

**Affiliations:** 1 Paperdrop Diagnostics S.L, MRB, Campus UAB, 08193 Bellaterra, Spain; 2 Nanobioelectronics and Biosensors Group, CSIC and BIST, Catalan Institute of Nanoscience and Nanotechnology (ICN2), Campus UAB, 08193 Bellaterra, Barcelona Spain; 3 Catalan Institution for Research and Advanced Studies (ICREA), Passeig de Lluís Companys, 23, 08010 Barcelona, Spain

**Keywords:** Rapid diagnostic tests, Lateral flow, Superbugs, Antimicrobial resistance, Point-of-care, Gold nanoparticles

## Abstract

Mainly performed within a rapid diagnostic tests company, a lateral flow (LF) system using gold nanoparticles (AuNPs) as transducers is presented able to detect three bacteria of interest, of relevance for antimicrobial resistance (AMR): *Clostridioides difficile*, methicillin-resistant *Staphylococcus aureus* (MRSA), and *Klebsiella pneumoniae*, with a limit of detection of 25 ng/mL of glutamate dehydrogenase (GDH) for *C. difficile*, 36 ng/mL of penicillin-binding protein 2a (PBP2a) for MRSA, and 4 × 10^6^ CFU/mL for *K*. *pneumoniae.* The system showed good results with bacteria culture samples, is user-friendly, and suitable for rapid testing, as the results are obtained within 15 min.

**Graphical Abstract:**

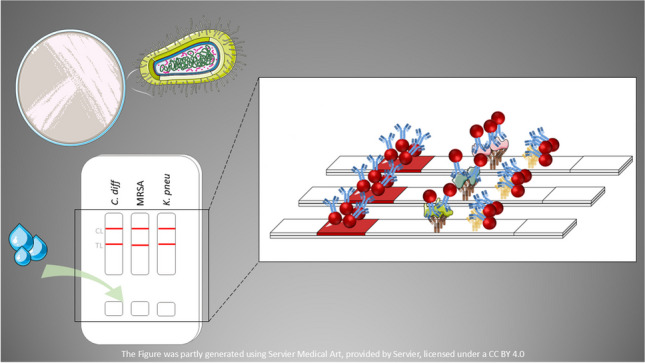

**Supplementary Information:**

The online version contains supplementary material available at 10.1007/s00604-024-06701-w.

## Introduction

Antimicrobial resistance (AMR) is defined by the World Health Organization (WHO) as the “ability of a microorganism – like bacteria, viruses and some parasites – to stop an antimicrobial (such as antibiotics, antivirals and antimalarials) from working against it” [1]. AMR is becoming a serious burden and threat to global public health [1–3]; according to the Center for Disease Control and Prevention (CDC), only in the USA, every year, more than 2.8 million antibiotic-resistant infections happen, and because of them, more than 35,000 people die [2]. Particularly concerning is the spread of multidrug-resistant bacteria, also known as “superbugs” [3].

One way to control this threat would be the use of cheap and portable biosensors [4], systems that make use of a biological element (e.g., an antibody), to rapidly identify superbugs spread. A popular example of biosensor is the rapid diagnostic tests (RDT), which have become very well-known during the last years due to their use for SARS-CoV-2 detection [5]. Most of the RDT employed were based on lateral flow (LF) technology [6–8], the same principle employed on pregnancy tests, because of their portability, low-cost production, and easiness of use. These characteristics allow LF tests to be used at point-of-care (PoC) [9], near the patient, or even in the environment (e.g. fomites [10] or contaminated water [11]), including hospital facilities. The use of nanomaterials in LF assays is well-known [12–16], since they permit increasing the sensitivity of the assay and reducing the limit of detection (LoD), allowing the development of LF tests for personalized medical applications [16–18].

Here, we present for the first time an LF system able to simultaneously detect three bacteria included in the list of antibiotic resistance threats of the CDC [2]: *Clostridioides difficile* (*C. diff.*), methicillin-resistant *Staphylococcus aureus* (MRSA), and *Klebsiella pneumoniae* (*K. pneu.*). The detection of these bacteria was also of interest within the framework of Anti-Superbugs pre-commercial procurement project [19], which had the objective to support research and development activities intended to identify these concrete three resistant micro-organisms, often responsible for hospital acquired infections (HAI), and control their spread.

**Fig. 1:**
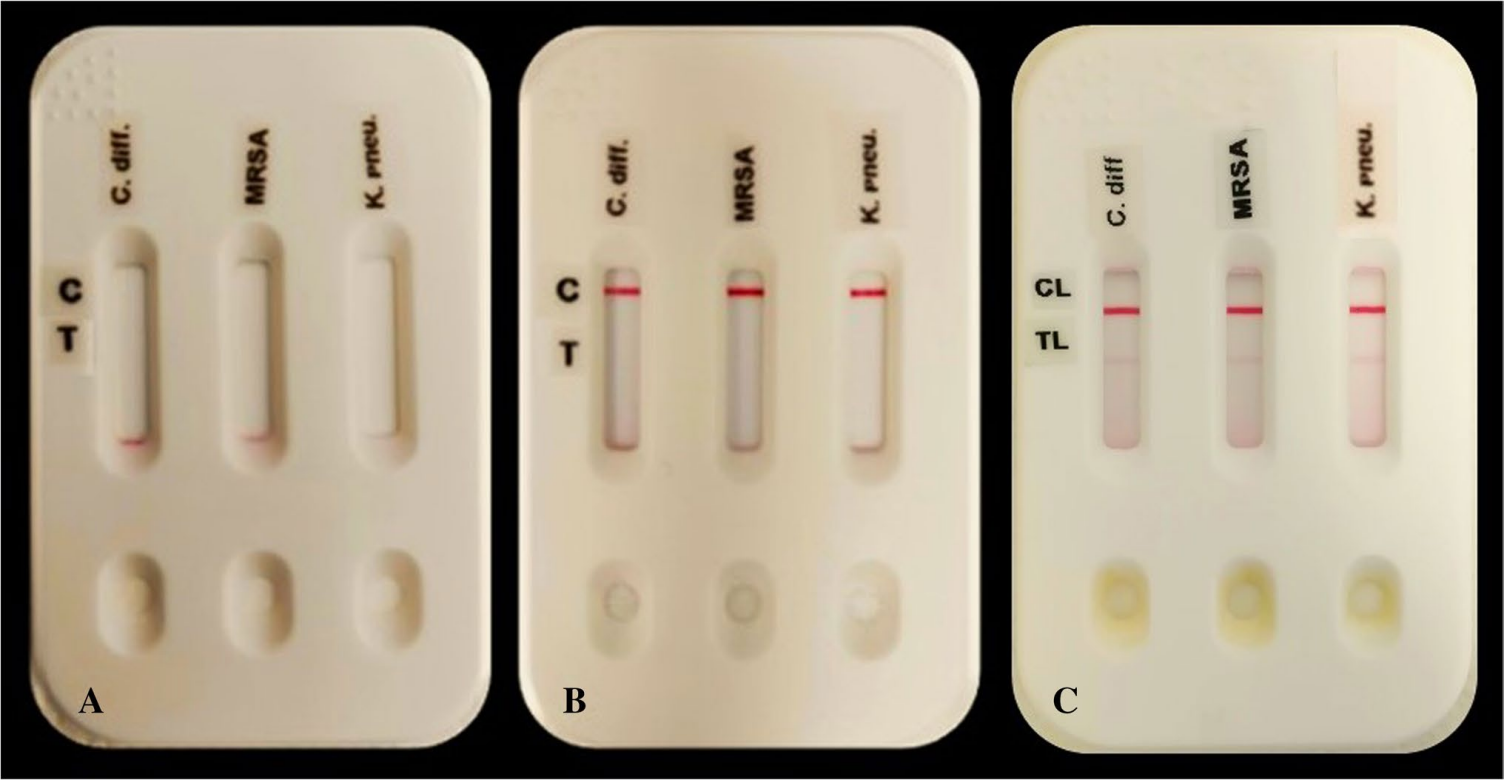
Our LF multiplexed system composed by a plastic cassette containing three independent LF strips, one for each target (from left to right: *C. diff.*, MRSA, and *K. pneu.*). **A** System before the testing. **B** System with a negative result for the three targets. **C** System with a positive result for the three targets

The system is provided in a plastic cassette, which holds inside three independent strips for carrying out a multiplexed test (Fig. 1). Gold nanoparticles (AuNPs) were used as label nanomaterial (conjugated to antibodies) due to the strong red color they can produce [7, 11, 12].

Each LF strip is based on immunochromatographic assays where, if the target is present, it will be captured between the antibody (Ab) in the AuNPs and the Ab in the test line (TL), leading to the formation of a red line in the TL position (as happened in Fig. 1C), besides the appearance of the control line (CL) (shown in both Figs. 1B and 1C). For the detection of *C. diff.*, antibodies against glutamate dehydrogenase (GDH) are used as recommended by the Spanish Society of Infectious Diseases and Clinical Microbiology [20]; in the case of MRSA detection, antibodies are specific for penicillin-binding protein 2a (PBP2a) [21, 22]; and lastly, for detection of *K. pneu.*, polyclonal antibodies against the bacteria are included [23]. Our test can be performed in just 15 min, and it is user-friendly (any person should be able to use it without any special training).

## Materials and methods

### Materials

HAuCl_4_, trisodium citrate, anhydrous sodium tetraborate, boric acid, sucrose, sodium dodecyl sulfate, bovine serum albumin, and Tween 20 are from Scharlab; tris-buffered saline tablets, phosphate-buffered saline tablets, Tergitol, and lysozyme are from Merck; cellulose fiber, laminated cards, and glass fiber are from Millipore; nitrocellulose membrane is from Pall Life Science; and cassettes are from Cangzhou ShengFeng Plastic Product Co., LTD. Summarized information about the employed antibodies (Ab) is compiled in Table 1; information about Ab suppliers and host species are restricted due to confidential requirements from Paperdrop Diagnostics S.L. (see conflicts of interest statement).

**Table 1:** Antibodies used in the multiplexed system and relevant information

Code	Target	Isotype	Clonality	Position in the test
Ab1	MRSA	Mouse IgG2b	Monoclonal	TL
Ab2	PBP2 from MRSA	Mouse IgG1	Monoclonal	AuNPs
Ab3	GDH from *C. diff*	Mouse IgG1	Monoclonal	AuNPs
Ab4	GDH from *C. diff*	Mouse IgG1	Monoclonal	TL
Ab5	*K. pneu*	Rabbit IgG	Polyclonal	TL
Ab6	*K. pneu*	Rabbit IgG	Polyclonal	AuNPs
Ab7	Anti-mouse	Goat IgG	Polyclonal	CL for MRSA and *C. diff.* assays
Ab8	Anti-rabbit	Goat IgG	Polyclonal	CL for *K. pneu*. assay

### Equipment

Thermoshaker (EQN022) is from Labnet; Biocen Centrifuge (model 22R) is from Orto Alresta; UV–Vis Spectrophotometer (SpectraMax iD3) is from Molecular Devices; Continuous Reagent Dispenser (HM88008) and Programmable Strip Cutter (ZQ2002) are from Shanghai Kinbio Tech; HP DeskJet Plus 4100 series scanner, Mestra oven (model 80,118), and autoclave (MED20) are from J.P Selecta; and TEM JEOL 1210 is located at the Institute of Materials Science of Barcelona.

### Gold nanoparticle synthesis and characterization

We followed the Turkevich method [24] to synthesize 20 nm diameter AuNPs. In brief, the synthetic procedure consists of 50 mL of 0.01% (w/v) HAuCl_4_ boiling solution and then quickly adding 1.25 mL of 1% (w/v) sodium citrate solution under vigorous stirring. The nanoparticle suspension color changes from colorless to purple and then red. When the suspension turns red, the temperature is turned off, and it is left to cool down while stirring. AuNPs suspension is stored in the fridge (never frozen).

Before using AuNPs batch, we characterized them by two different methods. First, by UV–Visible spectroscopy since 20 nm AuNPs exhibit a maximum absorbance peak at a wavelength of 520 nm (Figure S1) [25]. If the spectrum is as expected, then we characterize the nanoparticles by transmission electron microscopy (TEM) to verify there are no undesired structures and that shape and dimension are homogeneous (Figure. S2).

### Recombinant proteins and bacterial cultures

PBP2a recombinant protein (RayBiotech) was ordered as a target for the MRSA assay and resuspended to 1 mg/mL with sterile PBS. GDH recombinant protein (Medix Biochemica) was used as the target for *C. diff*. assay. The following bacteria cultures were ordered from the Spanish Collection of Type Culture (CECT): CECT 531, *Clostridioides difficile* (Hall and O'Toole 1935, Prévot 1938) Lawson et al. 2016, CECT 142Q: *Klebsiella pneumoniae subsp. pneumoniae* (Schroeter 1886) Trevisan 1887, CECT 5190: *Staphylococcus aureus subsp. aureus* Rosenbach 1884, CECT 794: *Staphylococcus aureus subsp. aureus* Rosenbach 1884. The mentioned cultures were obtained freeze-dried and were resuspended in 1 mL of PBS before use.

CECT 5190 corresponds to a MRSA culture and CECT 794 to a methicillin-sensitive *Staphylococcus aureus* (MSSA). CECT 142Q was quantified by CECT and had a concentration of 1.1 × 10^9^ CFU/mL. The other cultures were not quantified but had an estimated concentration in the order of 10^8^ CFU/mL.

### Lateral flow strip preparation

**Fig. 2:**
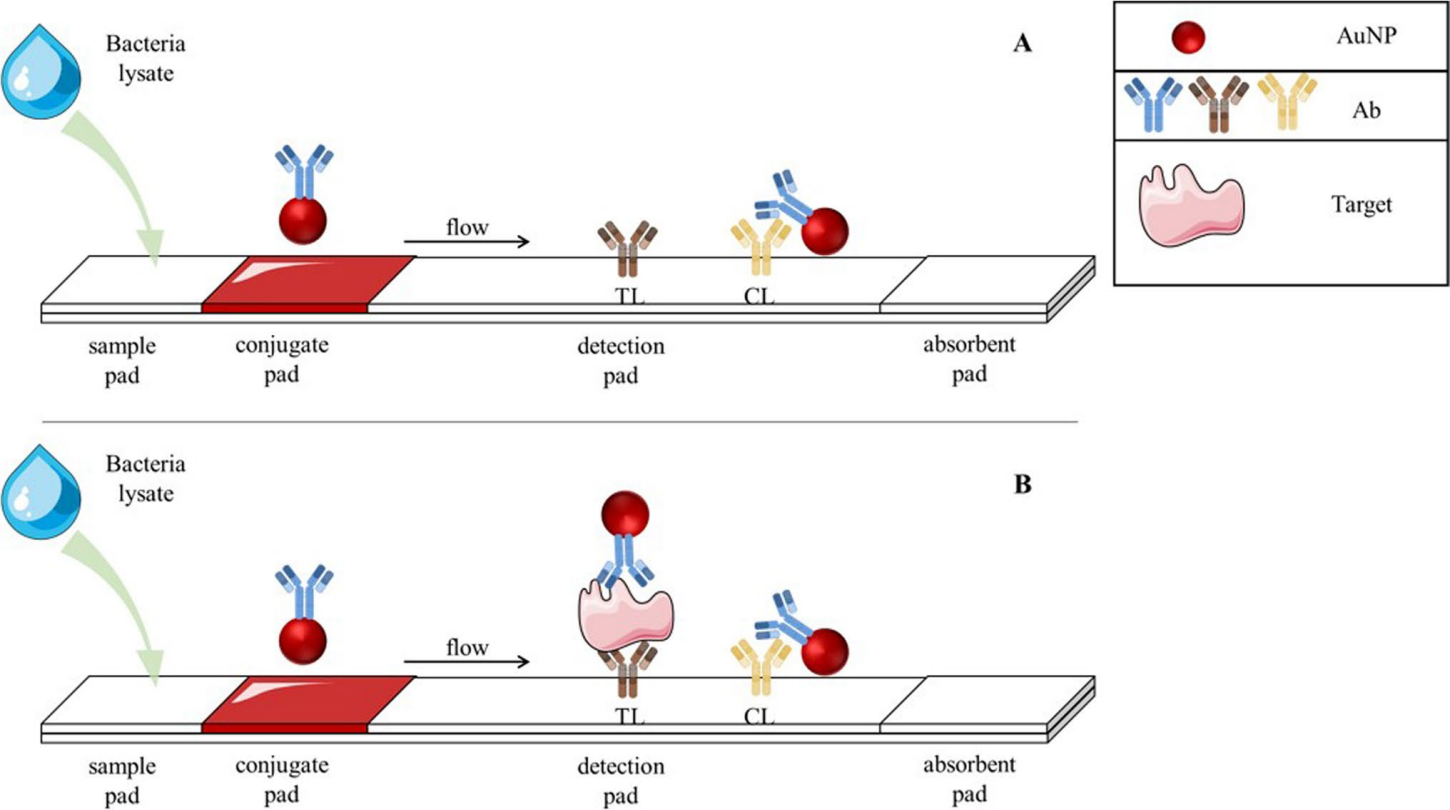
Schematic representation of the working principle of each LF strip. **A** When the target is not present, only the CL will appear. **B** If the target is present, it will be captured in a sandwich between the Ab of the TL and the Ab of AuNPs, leading to the appearance of the TL

Our system comprises three different LF strips, one for each bacterium; however, the methodology to construct the strips is the same otherwise indicated. An LF strip (Fig. 2) is composed by four pads (sample, conjugate, detection, and absorbent) which are assembled on a laminated adhesive card following a standard procedure [26]. All buffers employed in this section were autoclaved during 30 min at 121° before their use.

Briefly, the physical principle of this assay is based on capillarity action. When the extracted specimen is added to the sample pad, the flow goes through the paper reaching the conjugate pad, where the antibodies labeled with AuNPs are stored, ready to react to the target analyte (if present). Once the flow reaches the detection pad, if the specimen contains the target, the TL will appear indicating a positive result due to the presence of a specific Ab that recognizes the target (Fig. 2B). Therefore, if there is no target on the sample, the TL will not appear visible. The sample continues flowing through the detection pad and a CL, containing a secondary Ab, should appear for all valid tests (Fig. 2A). Finally, an absorbent pad is located at the end of the strip for absorbing the fluid excess.

Cellulose fiber is the main component of sample and absorbent pads, being only the first one pre-treated. To pre-treat the sample pad, it is immersed in PBS with 5% bovine serum albumin (BSA) and Tween 0.05% and then dried at 60 °C for 1 h.

AuNPs conjugated with antibodies are dry stored in the glass fiber (i.e., the conjugate pad). The conjugation protocol follows a procedure previously reported [26]. In brief, 1.5 mL of AuNPs previously adjusted to pH 9 using borate buffer 10 mM is mixed with 100 µL of 100 µg/mL antibody (see Table 1) and it is incubated during 30 min at 640 rpm at 20 °C. After this time, 100 µL of 0.1% (w/v) BSA solution is added to the conjugate, and the incubation is extended for additional 30 min. The conjugate is centrifuged at 18,000 rcf and 4 °C during 20 min to eliminate unconjugated antibodies, salts, and BSA (the supernatant is discarded), and the pellet is resuspended in 0.5 mL pH 7.5 borate buffer 1 mM containing 10% sucrose. The mixture is immediately dispensed on glass fiber and vacuum dried.

On nitrocellulose membrane (i.e., the detection pad), different antibodies are deposited as a TL or as a CL. The antibodies selected and deposited in each strip are indicated in Table 1. Both TL and CL were deposited with continuous reagent dispenser at a concentration of 1 mg/mL.

Finally, LF strips are cut to 4-mm width, and the three different strips are stored inside the cassette (Fig. 1).

### Lateral flow immunoassays

For each assay, serial dilutions of the corresponding target (GDH, PBP2a, *K. pneu.*) were made in lysis buffer (confidential recipe of Paperdrop Diagnostics S.L.). The same buffer was used as a blank. Even though the aim is to have a qualitative test, 120 µL of each dilution was tested by triplicate and after 15 min, the LF strips were analyzed for a quantitative analysis to calculate the limit of detection (LoD). The method consisted in scanning all the strips with a standard PC scanner in order to obtain an image to be analyzed using ImageJ software [27].

### Target detection in bacteria culture

Samples of bacteria cultures CECT 531 (*C. diff.*), CECT 5190 (MRSA), and CECT 142Q (*K. pneu.*) in lysis buffer were analyzed by triplicate using the corresponding LF strips assay. To evaluate possible cross-reaction between targets, samples of each bacteria culture were tested by triplicate in the other LF strips assays. In order to assess if the MRSA assay could differentiate between MRSA and MSSA, cultures of the two types of *S. aureus* (CECT 5190 vs CECT 794) were tested by triplicate.

Every time, 120 µL of each sample in lysis buffer was added to the sample pad, and results were analyzed after 15 min. Lysis buffer was used as a blank in each test.

## Results and discussion

### Lateral flow immunoassays

GDH was serial diluted in lysis buffer to the following concentrations: 10, 30, 50, 100, 200, 300, 500, 1000, and 3000 ng/mL. A total of 120 µL of the dilutions and blank (lysis buffer) were applied on the *C. diff.* LF strips, by triplicate.

PBP2a was serial diluted in lysis buffer to the following concentrations: 10, 30, 50, 100, 200, 1000, 2000, 3000, 5000, and 7000 ng/mL. A total of 120 µL of the dilutions and blank (lysis buffer) were applied on the MRSA LF strips, by triplicate.

Culture 142Q was serial diluted in lysis buffer to the following concentrations: 3.7 × 10^6^, 1.1 × 10^7^, 3.7 × 10^7^, 1.1 × 10^8^, and 3.7 × 10^8^ CFU/mL. A total of 120 µL of the dilutions and blank (lysis buffer) were applied on the *K. pneu.* LF strips, by triplicate.

**Fig. 3:**
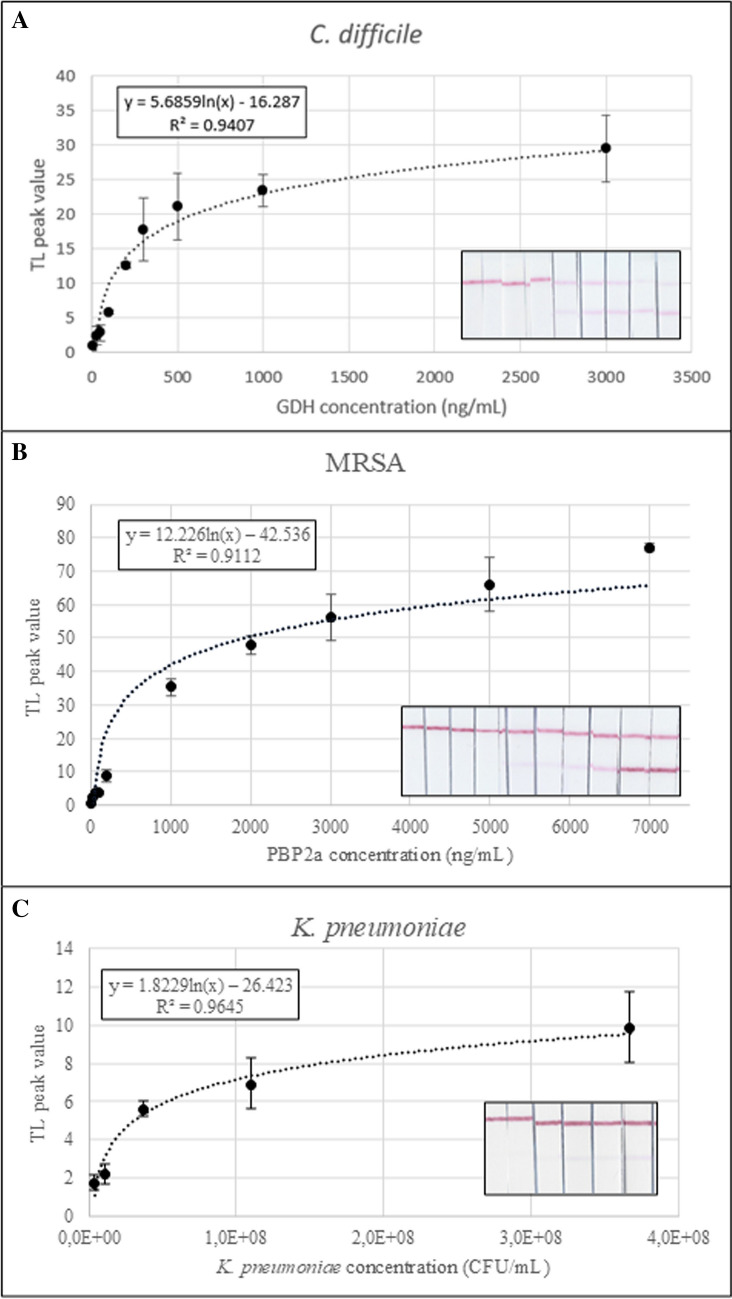
Calibration curve obtained for **A** *C. diff.*, **B** MRSA, and **C** *K. pneu*

The calibration curves obtained after analyzing the data acquired with a PC scanner and ImageJ [28] are represented in Fig. 3. The calculated equations obtained for each target are the following:

*y* = 5.6859 ln(x) − 16.287, *R*^2^ = 0.9407 (*C. diff.*)

*y* = 12.226 ln(x) − 42.536, *R*^2^ = 0.9112 (MRSA).

*y* = 1.8229 ln(x) − 26.423, *R*^2^ = 0.9645 (*K. pneu.*)

For each target, limit of detection (LoD) was calculated from the calibration equation by solving the “x” value when “y” is the “TL peak” value at blank (i.e., the average signal of blank, “b”) plus 3.3 times its standard deviation (σ) [29, 30]. The results were a LoD of 25 ng/mL of GDH for *C. diff.*, 36 ng/mL of PBP2a for MRSA, and 4 × 10^6^ CFU/mL for *K. pneu*. *C. diff.*, being a strict anaerobe, is technically difficult to quantify the culture, the reason why LoD is provided on ng/mL of GDH. In the case of MRSA, rapid tests in the market include LF assays [31] and latex agglutination tests [32–35], being in both cases the sample taken from a culture. The LoD of the commercialized LF is 7.3 × 10^8^ CFU/mL [36] and the required sample for the agglutination tests [37] are 1.5 × 10^9^ cells. We cannot compare directly with our calculated PBP2a LoD, but as shown in this work, this order of CFU/mL is being detected by our platform (culture samples had an estimated concentration in the order of 10^8^ CFU/mL).

### Target detection in bacteria culture

**Fig. 4:**
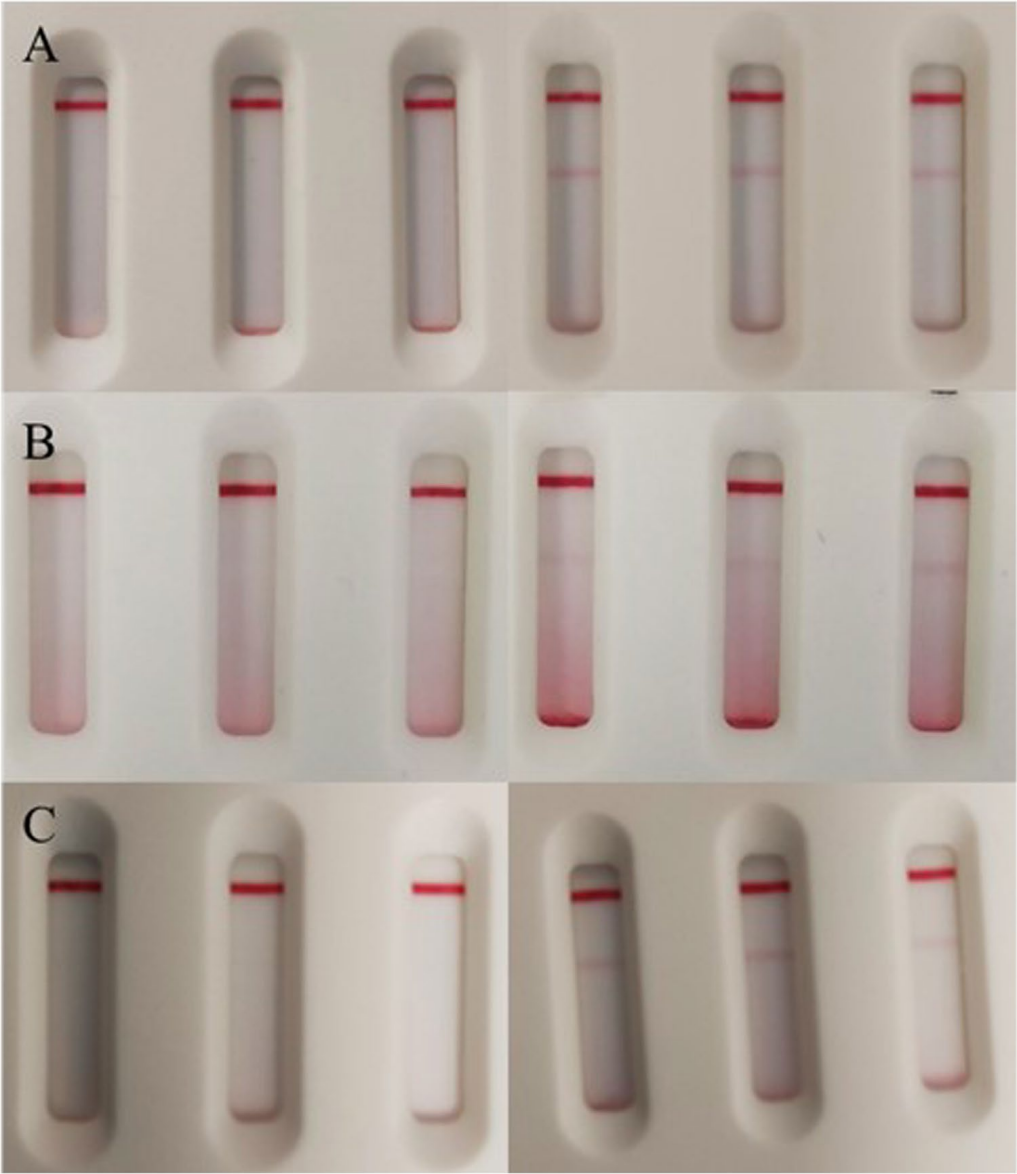
Visual response of the LF strips when testing bacterial culture of **A** *C. diff.*, **B** MRSA, and **C** *K. pneu.* For each target, three replicates of the negative (left) and positive (right) sample were tested

Samples of bacteria culture CECT 531 (*C. diff.*), CECT 5190 (MRSA), and CECT 142Q (*K. pneu.*) in lysis buffer were tested by triplicate using the corresponding LF strips assay. For CECT 531 and CECT 5190, 1:3 and 1:10 dilutions were tested. For CECT 142Q, several serial dilutions were tested as shown in the previous section. In each case, after 15 min, positive signals were observed that were distinguishable from the negative sample (Fig. 4), proving detection of the aimed bacteria.

No cross-reaction was observed when samples of CECT 531 (*C. diff.*), CECT 5190 (MRSA), and CECT 142Q (*K. pneu.*) in lysis buffer were tested with the LF strips which were not intended for them, as expected (Figure S3). When testing culture CECT 794 (MSSA) and CECT 5190 (MRSA), only positive signal was obtained with MRSA (Figure S4), indicating that the system can differentiate between the two *S. aureus*.

## Conclusions

In summary, a portable and equipment-free immunoassay system comprised by three LF strips devoted to the rapid detection of *C. diff.*, MRSA, and *K. pneu.* has been developed, being the first multiplexed test including those targets. The system reaches a LoD of 25 ng/mL of GDH for *C. diff.*, 36 ng/mL of PBP2a for MRSA, and 4 × 10^6^ CFU/mL for *K. pneu*. It has been demonstrated that the three LF strips can detect the target bacteria, due the good performance observed when testing bacterial culture. The presented system is a fast and easy method for the detection of the aforementioned bacteria that can be easily adapted for the identification of other superbugs. Bacteria identification is essential to control the transmission of multi-drug-resistant bacteria that can lead to hospital-acquired infections. More details about the use we propose for our system are shown in the video of reference [38] (BugWatcher Consortium, from Anti-SUPERBugs PCP Project [19]). In the video, readers can see that the test is employed to activate the alert protocol in the hospital areas when any of the three bacteria are identified, thus isolating the area and the patients. Every hospital may have different gold standard methods to later determine specific strains of the bacteria, if needed.

## Supplementary Information

Below is the link to the electronic supplementary material.

Supplementary file1 (PDF 442 KB)

## Data Availability

No datasets were generated or analysed during the current study.

## References

[1] World Health Organization (2020) Target product profiles for antibacterial resistance diagnostics. https://iris.who.int/bitstream/handle/10665/331054/9789240000407-eng.pdf?sequence=1&isAllowed=y. Accessed 30 May 2024

[2] Centers for Disease Control and Prevention, U.S. Department of Health and Human Services (2019) Antibiotic resistance threats in the United States. https://ndc.services.cdc.gov/wp-content/uploads/Antibiotic-Resistance-Threats-in-the-United-States-2019.pdf. Accessed 30 May 2024

[3] World Health Organization (2023) Antimicrobial resistance. https://www.who.int/news-room/fact-sheets/detail/antimicrobial-resistance. Accessed 30 May 2024

[4] Liu J, Mosavati B, Oleinikov AV, Du E (2019) Biosensors for detection of human placental pathologies: a review of emerging technologies and current trends. Transl Res 213:23–49 31170377 10.1016/j.trsl.2019.05.002 PMC6783355

[5] Drain PK (2022) Rapid Diagnostic Testing for SARS-CoV-2. N Engl J Med 386:264–272 34995029 10.1056/NEJMcp2117115 PMC8820190

[6] Van Amerongen A, Veen J, Arends HA, Koets M (2018) Lateral flow immunoassays. In: Handbook of immunoassay technologies: approaches, performances, and applications, Elsevier, pp 157–182

[7] Quesada-González D (2018) Design and application of nanomaterial-based lateral flow devices. Thesis dissertation. Autonomous University of Barcelona, Spain

[8] Jia X et al (2022) Highly sensitive detection of three protein toxins via SERS-lateral flow immunoassay based on SiO2@Au nanoparticles. Nanomed Nanotechnol, Biol Med 41:102522 10.1016/j.nano.2022.102522 35032631

[9] Quesada-González D, Merkoçi A (2018) Nanomaterial-based devices for point-of-care diagnostic applications. Chem Soc Rev 47:4697–4709 29770813 10.1039/c7cs00837f

[10] Ngu MAVN, Bergantin JH, Ramos JDA (2019) Development of a gold nanoparticle-labeled sandwich format lateral flow immunoassay kit for the detection of tropical house dust mite Suidasia pontifica. Protein Pept Lett 26:357–363 30760184 10.2174/0929866526666190212164751

[11] Quesada-González D, Jairo GA, Blake RC II, Blake DA, Merkoçi A (2018) Uranium (VI) detection in groundwater using a gold nanoparticle/paper-based lateral flow device. Sci Rep 8:8–15 30385866 10.1038/s41598-018-34610-5 PMC6212437

[12] Quesada-González D, Merkoçi A (2015) Nanoparticle-based lateral flow biosensors. Biosens Bioelectron 73:47–63 26043315 10.1016/j.bios.2015.05.050

[13] Quesada-González D et al (2019) Iridium oxide (IV) nanoparticle-based lateral flow immunoassay. Biosens Bioelectron 132:132–135 30870639 10.1016/j.bios.2019.02.049

[14] Huang Y et al (2020) Lateral flow biosensors based on the use of micro- and nanomaterials: a review on recent developments. Microchim Acta 187(1):70 10.1007/s00604-019-3822-x 31853644

[15] Rubio-Monterde A, Quesada-Gonzalez D, Merkoçi A (2023) Toward integrated molecular lateral flow diagnostic tests using advanced micro- and nanotechnology. Anal Chem 95(1):468–489 36413136 10.1021/acs.analchem.2c04529

[16] Napione L (2021) Integrated nanomaterials and nanotechnologies in lateral flow tests for personalized medicine applications. Nanomaterials 11(9):2362 34578678 10.3390/nano11092362 PMC8465858

[17] Toubanaki DK, Margaroni M, Prapas A, Karagouni E (2020) Development of a nanoparticle-based lateral flow strip biosensor for visual detection of whole nervous necrosis virus particles. Sci Rep 10:1–12 32300204 10.1038/s41598-020-63553-z PMC7162894

[18] Hsiao WWW et al (2021) Recent advances in novel lateral flow technologies for detection of COVID-19. Biosensors 11:1–26 10.3390/bios11090295 PMC8466143 34562885

[19] Anti-Superbugs PCP (2020) https://antisuperbugs.eu/about-the-project/. Accessed 30 May 2024

[20] Alcalá-Hernández L, Mena-Ribas A, Niubó-Bosh J, Marín-Arriaza M (2016) Diagnóstico microbiológico de la infección por *Clostridium difficile*. Lab Diagn Clostridium Difficile Infect 34(9):595–602. 10.1016/j.eimc.2015.09.004 10.1016/j.eimc.2015.09.004 26493356

[21] Fishovitz J, Hermoso JA, Chang M, Mobashery S (2014) Penicillin-binding protein 2a of methicillin-resistant Staphylococcus aureus. IUBMB Life 66:572–577 25044998 10.1002/iub.1289 PMC4236225

[22] Matsui H et al (2011) Development of an immunochromatographic strip for simple detection of penicillin-binding protein 2′. Clin Vaccine Immunol 18:248–253 21177917 10.1128/CVI.00252-10 PMC3067364

[23] Tominaga T (2018) Rapid detection of *Klebsiella pneumoniae*, *Klebsiella oxytoca*, *Raoultella ornithinolytica* and other related bacteria in food by lateral-flow test strip immunoassays. J Microbiol Methods 147:43–49 29522975 10.1016/j.mimet.2018.02.015

[24] Turkevich J, Stevenson PC, Hillier J (1951) A study of the nucleation and growth processes in the synthesis of colloidal gold. Discuss Faraday Soc 11:55–75

[25] Barnes WL, Dereux A, Ebbesen TW (2003) Surface plasmon subwavelength optics. Nature 424:824–830 12917696 10.1038/nature01937

[26] Quesada-González D et al (2019) Signal enhancement on gold nanoparticle-based lateral flow tests using cellulose nanofibers. Biosens Bioelectron 141:111407 31207571 10.1016/j.bios.2019.111407

[27] Rivas L et al (2018) A vertical flow paper-microarray assay with isothermal DNA amplification for detection of Neisseria meningitidis. Talanta 183:192–200 29567164 10.1016/j.talanta.2018.02.070

[28] Parolo C et al (2020) Tutorial: design and fabrication of nanoparticle-based lateral-flow immunoassays. Nat Protoc 15:3788–3816 33097926 10.1038/s41596-020-0357-x

[29] Armbruster DA, Pry T (2008) Limit of blank, limit of detection and limit of quantitation. Clin Biochem Rev 29(Suppl1):S49-52 18852857 PMC2556583

[30] Moulahoum H, Ghorbanizamani F (2024) The LOD paradox: when lower isn’t always better in biosensor research and development. Biosens Bioelectron 264:116670 39151260 10.1016/j.bios.2024.116670

[31] Clearview PBP2a | Abbott point of care. https://www.globalpointofcare.abbott/en/product-details/clearview-pbp2a.html. Accessed 28 Aug 2024

[32] Louie L, Matsumura SO, Choi E, Louie M, Simor AE (2000) Evaluation of three rapid methods for detection of methicillin resistance in Staphylococcus aureus. J Clin Microbiol 38(6):2170–2173 10834971 10.1128/jcm.38.6.2170-2173.2000 PMC86755

[33] Hussain Z, Stoakes L, Garrow S, Longo S, Fitzgerald V, Lannigan R (2000) Rapid detection of MecA-positive AndmecA-negative coagulase-negative Staphylococci by an anti-penicillin binding protein 2a slide latex agglutination test. J Clin Microbiol 38(6):2051–2054 10834952 10.1128/jcm.38.6.2051-2054.2000 PMC86725

[34] Bressler AM, Williams T, Culler EE, Zhu W, Lonsway D, Patel JB, Nolte FS (2005) Correlation of penicillin binding protein 2a detection with oxacillin resistance in Staphylococcus aureus and discovery of a novel penicillin binding protein 2a mutation. J Clin Microbiol 43(9):4541–4544 16145104 10.1128/JCM.43.9.4541-4544.2005 PMC1234091

[35] Cavassini M, Wenger A, Jaton K, Blanc DS, Bille J (1999) Evaluation of MRSA-screen, a simple anti-PBP 2a slide latex agglutination kit, for rapid detection of methicillin resistance in Staphylococcus aureus. J Clin Microbiol 37(5):1591–1594 10203531 10.1128/jcm.37.5.1591-1594.1999 PMC84841

[36] FDA (2014) 510(k) Substantial equivalence determination decision summary assay - K133851. https://www.fda.gov/regulatory-information/search-fda-guidance-documents/510k-program-evaluating-substantial-equivalence-premarket-notifications-510k. Accessed 28 Aug 2024

[37] Thermo Fisher. Penicillin-binding protein (PBP2) latex agglutination test. https://www.thermofisher.com/order/catalog/product/DR0900A. Accessed 28 Aug 2024

[38] Bugwatcher (2021) BUGWATCHER, an ICT digital solution against Super-bugs. https://www.youtube.com/watch?v=7IVNFnhVUrE. Accessed 21 Aug 2024

