# Supplementary material for: Lateral flow immunoassay for simultaneous detection of C. difficile, MRSA, and K. pneumoniae
Source: Mikrochim Acta. 2024 Oct 1;191(10):638. doi: 10.1007/s00604-024-06701-w (PMC11445331; doi:10.1007/s00604-024-06701-w)
Supplement: Supplementary file 1 — Supplementary file1 (PDF 442 KB) [file 604_2024_6701_MOESM1_ESM.pdf]

Lateral flow immunoassay for simultaneous detection of *C.difficile*, MRSA and *K.pneumoniae*

Ana Rubio-Monterde[1,2] · Lourdes Rivas[1] · Marc Gallegos[1] · Daniel Quesada-González[1,2]*, Arben Merkoçi[1,2,3]*

[1] Paperdrop Diagnostics S.L., MRB, Campus UAB, 08193 Bellaterra, Spain

[2] Nanobioelectronics and Biosensors Group, Catalan Institute of Nanoscience and Nanotechnology (ICN2), CSIC and BIST, Campus UAB, Bellaterra, 08193 Barcelona, Spain

[3] Catalan Institution for Research and Advanced Studies (ICREA); Passeig de Lluís Companys, 23, Barcelona, 08010, Spain.

*Daniel Quesada-González – daniel.quesada@icn2.cat

*Arben Merkoçi – arben.merkoci@icn2.cat

UV-Visible spectroscopy AuNPs

3 spectra were measured from the synthetized AuNPs (3 aliquots). The maximum absorbance peak is detected for a wavelength of 519-520 nm. Therefore, the 3 spectra are considered satisfactory.

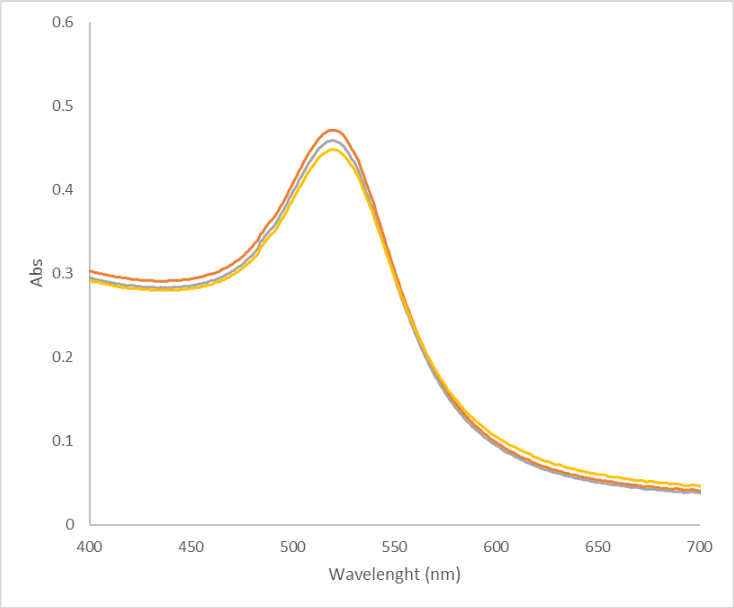

Fig. S1 Characterization by UV-Visible spectroscopy of the AuNPs synthesized

TEM AuNPs

TEM measurement of the synthetized AuNPs

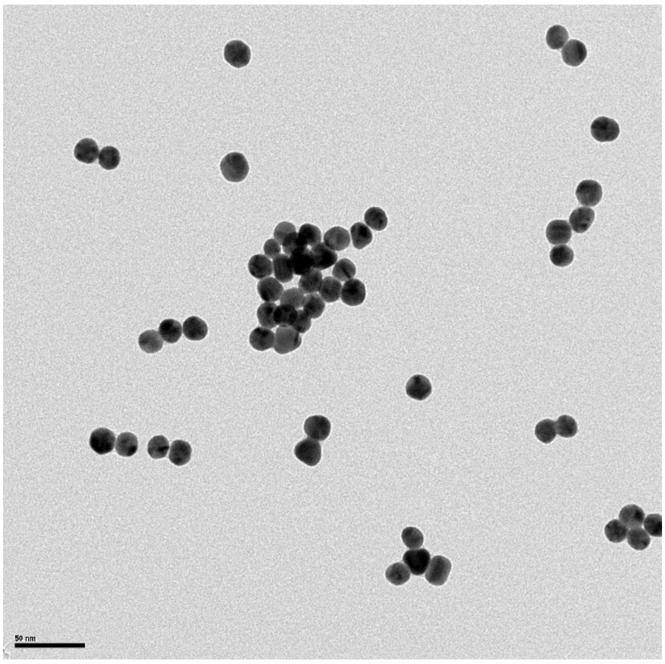

Fig. S2 Characterization by TEM of the AuNPs synthesized

Cross-reaction

To evaluate possible cross-reaction between the targets, triplicates of each strip assay were tested with the other 2 bacteria of the system. 1/10 dilution of CECT 531 (*C.diff*) and CECT 5190 (MRSA) and 1.1x10^7 CFU/mL of CECT 142Q (*K.pneu*) in lysis buffer were tested. No cross-reaction was observed when the samples were tested with the opposite LF strips - the assays not intended to detect that bacterium - (Figure S3).

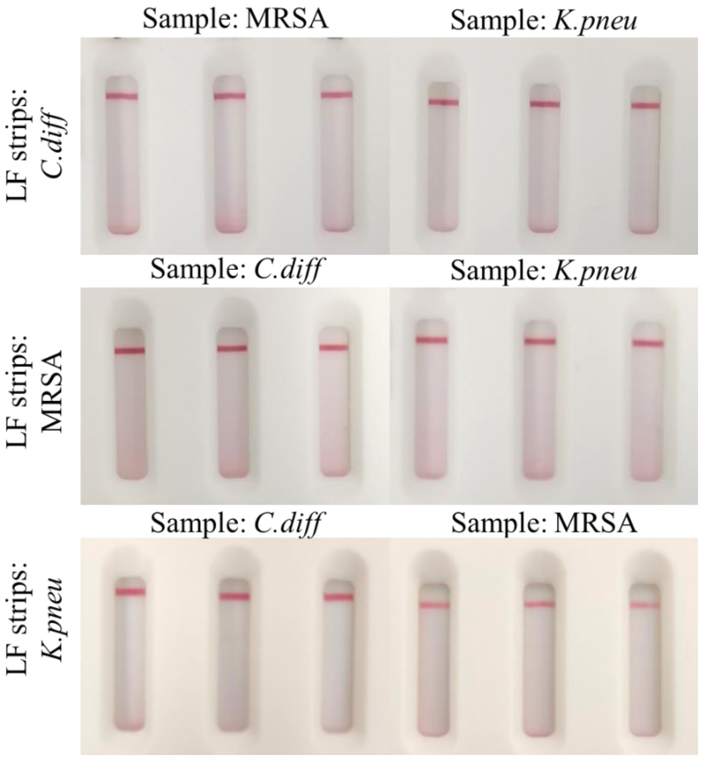

Fig. S3 Results of cross-reaction study. No unspecific signals observed

MSSA vs MRSA

Triplicates of the LF strip for MRSA were tested with culture of MSSA (CECT 794) and MRSA (CECT 5190). When testing the same dilution of culture CECT 794 (MSSA) and CECT 5190 (MRSA), only positive signal was obtained with MRSA (Figure S4), indicating that the system can differentiate between the two *S.aureus*.

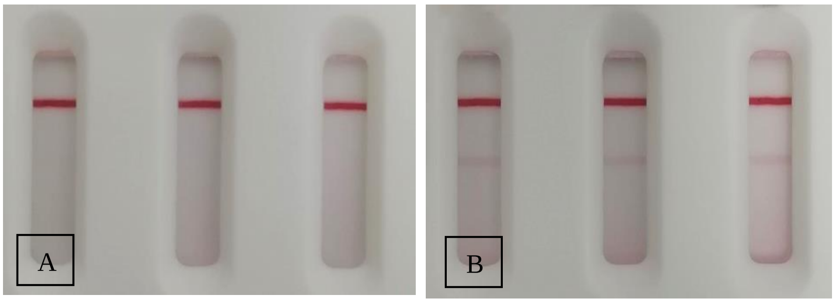

Fig. S4 Testing of MSSA (A) vs MRSA (B). No positive signal is observed when MSSA is tested but positive signal is shown with MRSA
